# Trans‐Specific Polymorphisms Between Cryptic *Daphnia* Species Affect Fitness and Behavior

**DOI:** 10.1111/mec.17632

**Published:** 2024-12-24

**Authors:** Connor S. Murray, Madison Karram, David J. Bass, Madison Doceti, Dörthe Becker, Joaquin C. B. Nunez, Aakrosh Ratan, Alan O. Bergland

**Affiliations:** ^1^ Department of Biology University of Virginia Charlottesville Virginia USA; ^2^ Department of Genome Sciences University of Virginia School of Medicine Charlottesville Virginia USA; ^3^ School of Biosciences, Ecology and Evolutionary Biology University of Sheffield Sheffield UK; ^4^ Department of Biology University of Vermont Burlington Vermont USA

**Keywords:** balancing selection, convergent evolution, *Daphnia*, hybridization, opsins, shared polymorphism

## Abstract

Shared polymorphisms, loci with identical alleles across species, are of unique interest in evolutionary biology as they may represent cases of selection maintaining ancient genetic variation post‐speciation, or contemporary selection promoting convergent evolution. In this study, we investigate the abundance of shared polymorphism between two members of the *Daphnia pulex* species complex. We test whether the presence of shared mutations is consistent with the action of balancing selection or alternative hypotheses such as hybridization, incomplete lineage sorting or convergent evolution. We analyzed over 2,000 genomes from six taxa in the *D. pulex* species group and examined the prevalence and distribution of shared alleles between the focal species pair, North American and European *D. pulex* . We show that North American and European *D. pulex* diverged over 10 million years ago, yet retained tens of thousands of shared polymorphisms. We suggest that the number of shared polymorphisms between North American and European *D. pulex* cannot be fully explained by hybridization or incomplete lineage sorting alone. We show that most shared polymorphisms could be the product of convergent evolution, that a limited number appear to be old trans‐specific polymorphisms, and that balancing selection is affecting convergent and ancient mutations alike. Finally, we provide evidence that a blue wavelength opsin gene with trans‐specific polymorphisms has functional effects on behavior and fitness in the wild.

## 1 Introduction

Genetic diversity reflects a species' history and serves as the foundation for adaptation to ecological change. In nature, mutations arise and their persistence time is a function of their selective value and the effective population size of the focal species (Li and Nei 1977), as well as features of the environment (Cvijović et al. 2015). One distinct type of genetic variant is a shared polymorphism, in which mutations are identical by state across closely related species (Wang and Mitchell‐Olds 2017). The abundance and frequency of shared polymorphisms between two species can provide insight into some of the most interesting processes in evolution. A shared polymorphism that arose prior to the split of two species is generally referred to as a trans‐species polymorphism (Hedrick 2013; Wiuf et al. 2004; Wu et al. 2017). Trans‐specific polymorphisms can be used to study the speciation process (Klein et al. 1998), helping refine estimates of the timing and population sizes at divergence (Edwards and Beerli 2000). Unless divergence happens recently or there is ongoing gene‐flow, it is unlikely that neutral polymorphisms will be retained in both species for long (Leffler et al. 2013). Therefore, the presence of trans‐species polymorphism between two species with limited gene‐flow can be a powerful way to identify balanced polymorphisms (Clark 1997). These polymorphisms are presumed to be maintained by temporal or spatial variation in the direction of natural selection or by genetic overdominance (e.g., Bergland et al. 2014; Cho et al. 2006; Cornetti et al. 2024; Wills 1975; Schield et al. 2022; Ségurel et al. 2012; Ségurel, Gao, and Przeworski 2013). Shared polymorphisms can also indicate convergent adaptive evolution (Castoe et al. 2009) or adaptive introgression (Hedrick 2013), and these polymorphisms themselves can also be the target of balancing selection (Wang and Mitchell‐Olds 2017).

Identifying the forces that generate and maintain shared polymorphism is therefore an important problem in evolutionary genetics. However, identifying the contribution of demographic and adaptive evolutionary processes to the generation and maintenance of shared polymorphisms is challenging. Testing alternative hypotheses for the generation of shared polymorphism is a tractable problem for older species pairs because neutral trans‐species polymorphisms are expected to be rare thereby eliminating incomplete lineage sorting as a possible explanation (Rosenberg 2003). If sufficient time has occurred for the fixation of species‐specific alleles, patterns of adaptive introgression can be identified (e.g., Huerta‐Sánchez et al. 2014; Setter et al. 2020), especially if it occurred recently. Evidence for long‐term balancing selection of a trans‐specific polymorphism becomes stronger if there are multiple sites at a locus that are shared, and these are tightly linked causing gene‐trees to group by haplotype and not by species (Wang et al. 2020). Moreover, the presence of trans‐specific haplotypes suggests that multiple functional sites at the locus are the target of balancing selection (Charlesworth 2006).

*Daphnia* are an excellent model to study the mechanisms that generate and maintain shared polymorphism. *Daphnia* are freshwater microcrustaceans that have been the focus of ecological and evolutionary research for over a century (Ebert 2022). Amongst the most widely studied taxa within this genus are *Daphnia magna* (Decaestecker et al. 2007), *Daphnia obtusa* (Spitze 1993), as well as *Daphnia pulex* (Lynch et al. 2017) and its close relatives (Colbourne et al. 2011). The *D. pulex* species group is currently undergoing adaptive radiation (Fryer 1991). Owing to their recent divergence, some members of the North American *D. pulex* species group are known to hybridize (Held, Koenemann, and Schubart 2016), resulting in introgression‐induced obligate asexuality (Xu et al. 2015) and the loss of male production (Ye et al. 2019). Members of the *D. pulex* species group, including *D. obtusa, D. pulicaria* and *D. pulex* , are found across the Palearctic and Nearctic (Crease et al. 2012), and recently established populations can be found in other regions of the world as invasives (So et al. 2015). Although *D. obtusa* , *D. pulicaria* and *D. pulex* have been identified on multiple continents, each of these taxa represents polyphyletic groups (Černý and Hebert 1999). For instance, *D. pulex* found in North America are more closely related to North American *D. pulicaria* than to European *D. pulex* (Crease et al. 2012). The confusion of species identification and naming in this genus is due to their similar morphology (Dodson 1981) and ecological niches (Chin and Cristescu 2021) plus their capacity to interbreed (Pantel, Juenger, and Leibold 2011), generally reflecting the taxonomic ambiguities within the species group (Hebert and Wilson 1994), and amongst zooplankton in general (Brooks 1957a, 1957b).

The evolutionary and ecological history of the *D. pulex* species group affords us an ideal opportunity to study the evolutionary forces that have shaped patterns of shared polymorphism. Here, we assessed alternative causes of shared polymorphisms between North American and European *D. pulex* . Using population genomic data, we first confirm that North American and European *D. pulex* are distinct species that diverged millions of years ago. Next, we show that North American and European *D. pulex* possess tens of thousands of shared polymorphisms, whose abundance cannot be explained by neutral, demographic models alone. Therefore, we conclude that many of these shared polymorphisms arose either via convergent evolution or have been maintained by selection since the split between these taxa. We identify a blue wavelength opsin with an excess of shared non‐synonymous polymorphisms that are in linkage disequilibrium, consistent with long‐term balancing selection operating on functionally distinct haplotypes. We show that European *D. pulex* clones harbouring alternate genotypes for this blue wavelength opsin have differences in movement and activity that are dependent on light conditions and provide evidence for overdominance in the wild. Taken together, our results highlight the abundance, selective history and function of shared polymorphisms in *Daphnia* and contribute to our understanding of the phylogeography for this model system.

## 2 Materials and Methods

### 2.1 Sampling and Sequencing of New European *Daphnia* Genomes

*Daphnia* were sampled from 16 ponds throughout England in 2018 (Table S1). Samples were transported to the University of Virginia and clonally derived isofemale lines were established. Samples were identified as either *D. pulex* , *D. pulicaria* or *D. obtusa* based on morphological characteristics using an online dichotomous key (http://cfb.unh.edu/cfbkey/html/anatomy/daphnia/daphnia.html). DNA extraction and library preparation followed methods outlined in Barnard‐Kubow et al. (2022). Briefly, isofemale lines were exposed to antibiotics (streptomycin, tetracycline and ampicillin, 50 mg/L of each) and fed Sephadex G‐25 beads to clear their gut of algae. Samples were homogenised using metal beads and a bead beater and DNA was extracted using the Agencourt DNAdvance kit (Beckman‐Coulter). RNA was removed using RNase. DNA was quantified using the broad‐range Quant‐iT dsDNA kit (ThermoFisher Scientific) and normalized to 1–2 ng/μL before library construction. Full genome libraries were constructed using a scaled‐down Nextera protocol (Baym et al. 2015). Libraries were size selected for fragments ranging from 450 to 550 bp using a Blue Pippin and quality checked using a BioAnalyzer. Samples were sequenced on a HiSeq X platform, paired‐end 150 bp.

### 2.2 Publicly Available *Daphnia* Genomes

Genome sequences of North American and European *D. pulex* , *D. pulicaria* and *D. obtusa* were obtained from NCBI's Sequence Read Archive (Leinonen et al. 2011). Additionally, we obtained short‐read sequence data for known hybrids between North American *D. pulex* and *D. pulicaria* . We incorporated wild‐sequenced or isogenic female lineages (Alzbutas 2015; Barnard‐Kubow et al. 2022; Jackson et al. 2021; Jiang et al. 2017; Lack 2017; Lynch et al. 2017; Maruki 2018; Maruki, Ye, and Lynch 2022; Tucker et al. 2013; Xu et al. 2015; Ye et al. 2022), and excluded samples that were from mutation accumulation studies. Species identity for these samples was based on annotations provided in each sequence read archive record. Information about each clone can be found in Table S1.

### 2.3 Short‐Read Mapping

Prior to mapping samples, adaptors were removed using *trimmomatic v0.39* (Bolger, Lohse, and Usadel 2014), and overlapping reads were merged using *pear v0.9.11* (Zhang et al. 2014). All samples were mapped to the European *D. pulex* genome (D84A; GenBank assembly: GCA_023526725.1; Barnard‐Kubow et al. 2022) using *bwa mem v0.7.17* (Li 2013), and downstream data manipulation was performed using *samtools merge v1.12* (Li et al. 2009). Duplicate reads were marked and removed using *picard v2.23.4* (https://broadinstitute.github.io/picard/).

Additionally, we mapped North American *D. pulex* to the North American *D. pulex* reference genome (KAP4; GenBank assembly: GCF_021134715.1; Ye, Pfrender, and Lynch 2023) using the same mapping strategy outlined above. We created a liftOver file to translate features in KAP4 to D84A. We created the liftOver file by running pairwise alignments using *lastz v1.04.22* followed by the use of various UCSC tools to chain the alignments, sort them, filter them and convert them into UCSC nets and chains (Harris 2007). The chains exhibited good coverage, allowing us to translate 72.6% of the KAP4 genome to D84A (Lee et al. 2022). We used *LiftOverVCF* from *picard v2.23.4* to convert the KAP4‐aligned VCF to the D84A genome coordinates.

### 2.4 SNP Calling and Filtering

We performed SNP calling using *HaplotypeCaller* and *GenotypeGVCFs* from *gatk v4.1.6.0* (Poplin et al. 2017). We called SNPs by first using the short‐reads for all taxa in the *D. pulex* species group mapped to the European *D. pulex* genome (D84A). Additionally, we called SNPs in North American *D. pulex* using the reads mapped back to the North American *D. pulex* genome (KAP4). In both strategies, we used *VariantFiltration* in *gatk* to remove low‐quality SNPs recommended for organisms without reference panels: (‘QD < 2.0’, ‘QUAL < 30.0’, ‘SOR > 3.0’, ‘FS > 60.0’, ‘MQ < 40.0’, ‘MQRankSum < −12.5’ and ‘ReadPosRankSum < −8.0’). We removed sites flanking indels (±10 bp) using *bcftools filter* (Li et al. 2009) and removed indels. We annotated SNPs using *snpEff v4.3t* (Cingolani et al. 2012).

We filtered the dataset by first removing all samples with average genome‐wide coverage < 8×. Next, we filtered any genomic region (10 kbp window) with high (95% upper quantile tail; ≥ 35×) or low mean site read coverage (5% lower quantile; ≤ 8×) across all samples, along with chromosomal endpoints and regions ±500 bps surrounding unassembled gaps that likely represent transposable elements. Repetitive elements identified in both the European and North American *D. pulex* genomes were classified with *RepeatMasker v4.0.8* and removed (Tarailo‐Graovac and Chen 2009). Most analyses removed SNPs that have a minor allele frequency (MAF) < 0.01 within‐species. After this initial filtering, 347,200 SNPs remained, representing the whole‐genome SNP set. For the principal component, D/f4 and admixture analyses, we used the whole‐genome SNP set.

We performed additional filtering of the SNP set for different analyses to produce a conservative dataset. For the phylogenetic analysis, we used the SNP set generated by mapping all genomes to the European *D. pulex* reference genome and restricted analysis to SNPs surrounding BUSCO genes (Mi et al. 2013; Seppey, Manni, and Zdobnov 2019; Simão et al. 2015). This multi‐species BUSCO gene SNP set includes 138,024 SNPs. For the analyses of cophenetic distances, balancing selection and individual genes (e.g., see below BLOP gene), we restricted analyses to genic (3′UTR, 5′UTR, exon and intron) and non‐genic (downstream and upstream up to 5 kbps) regions associated with the 6,544 single‐copy ortholog genes between European *D. pulex* and North American *D. pulex* identified by *OrthoFinder v5* (Emms and Kelly 2019) for the SNPs that were retained from the liftOver. Additionally, we only used the SNPs classified in the same manner (i.e., within‐species polymorphisms, shared polymorphisms or fixed differences) when mapping to either European and North American *D. pulex* assembly (Figure S1A,B). We show that 88% of SNP classifications are unchanged between assemblies (Figure S1B,C; *N* = 28,983; Table S2).

A concern for aligning divergent sequences to the same assembly is for reference allele bias to decrease mapping efficiency and cause genotype errors (Günther and Nettelblad 2019). To assess this, we calculated the proportion of alternative and reference allele dosage for heterozygous BUSCO gene SNPs (*N* = 1,000; 100 bootstraps). On average, SNPs identified in North American or European *D. pulex, D. pulicaria* or *D. obtusa* had approximately the same alternative and reference allele dosage at heterozygous sites, revealing an absence of systematic reference allele bias (Figure S1D). Therefore, we conclude that the data are not systematically biased by mapping reads from non‐European *D. pulex* to the European *D. pulex* assembly.

### 2.5 Assigning Multi‐Locus Genotypes

Every sample was assigned to a multi‐locus genotype (MLG) using the *poppr v2.9.3* package (Kamvar, Tabima, and Grünwald 2014) in *R v4.0.3* using the genome‐wide SNP set (*N* = 347,200). Unless otherwise noted, every analysis was subset based on picking a representative sample with the highest coverage for each MLG.

### 2.6 Mitochondrial Tree

We annotated the D84A mitochondrial genome using *MITOs v1* (Bernt et al. 2013). We aligned and called SNPs using *bcftools mpileup v1.9* and *bcftools call*. We excluded reads that had low‐quality scores (*Q* < 20) and high coverage (DP > 100) using *bcftools filter*, and generated consensus FASTA files using *bcftools consensus*. We mapped North American *D. pulex* and *D. pulicaria* to the North American *D. pulex* mitochondrial genome (GenBank accession: NC_000844.1) and mapped both North American and European *D. obtusa* samples to the North American *D. obtusa* mitochondrial genome (GenBank accession: CM028013.1). The mitochondrial sequence of European *D. magna* was used as an outgroup (GenBank accession: NC_026914.1). We assessed sequence similarity using *exonerate v2.4.0* (Slater and Birney 2005) for the 13 protein‐coding genes and found high sequence similarity (> 80%), except for *atp8*. Therefore, we assembled trees excluding *atp8*. We then used *mafft v7.475* (Katoh and Standley 2013) to assemble multiple sequence alignments (MSA). We concatenated these MSAs for each gene using *seqkit concat v2.2.0* (Shen et al. 2016) and ran *iqtree2 v2.1.2* with 1,000 bootstraps (Figure S2; Hoang et al. 2018).

### 2.7 Estimating Divergence‐Time

We used *Snapp v1.6.1* within *Beast2 v2.6.6* to estimate the split‐time between‐species (Bouckaert et al. 2014). We used two representative individuals with the highest coverage for each species. We used 3,000 randomly sampled BUSCO SNPs, one million iterations and a 10% burn‐in. The output tree was time‐constrained for the outgroup species, European *D. obtusa* , to 31 million years ago (mya with a confidence interval of 1 mya based upon a genus‐wide tree; Chin and Cristescu 2021; Cornetti et al. 2019). We used *Tracer v1.7.1* to quantify convergence and set the generation time to one per year (Rambaut et al. 2018).

### 2.8 Hybridization Statistics

We used *ADMIXTURE v1.3.0* (Alexander and Lange 2011), excluding any sites with MAF < 0.01 and thinned every 500 SNPs. We varied the number of *k‐*clusters and calculated the cross‐validation error for every value of *k*. We quantified the magnitude of introgression using *Dsuite v0.5* (Malinsky, Matschiner, and Svardal 2021) with European *D. obtusa* as the outgroup.

### 2.9 Historic *N*_e_, Neutral Models of Incomplete Lineage Sorting and Demographic Inference of Migration

To calculate historic *N*_e_ for European and North American *D. pulex* , we ran *MSMC2 v2.1.1* (Schiffels and Wang 2020) with a fixed recombination rate and the ‐‐timeSegmentPattern parameter set to 10 * 1 + 15 * 2 following Nunez et al. (2021). We ran *eSMC v2.0.5* (Sellinger et al. 2020), a software designed for facultatively sexual organisms to estimate historic *N*_e_ and the hatching rate from resting eggs per generation (𝛽). *eSMC* was run by chromosome and we scaled the recombination rate of 8 × 10^−8^ per generation by 5 to account for five generations of parthenogenesis prior to a hatching event as described in Sellinger et al. (2020).

We tested whether the extent of shared polymorphism can be explained by incomplete lineage sorting (Novikova et al. 2016; Wiuf et al. 2004). The formula in Novikova et al. (2016) uses the expected coalescence times within and between‐species to estimate the upper bound of the number of shared polymorphisms at synonymous sites given no migration. This method uses the level of polymorphism within‐species to reflect the within‐species coalescent time and assumes that the population size (and thus diversity level) of the ancestral species is the same as the larger of the two diversity estimates of the extant species. See Novikova et al. (2016) for a more in‐depth derivation of the method. The upper bound of the number of shared polymorphisms given incomplete lineage sorting is

$$\exp\left(-2d_{\text{between}}+\frac{\left(p_{\mathrm{NAm}}+p_{\text{Euro}}\right)\max\left\{p_{\mathrm{NAm}},p_{\text{Euro}}\right\}}{p_{\mathrm{NAm}},p_{\text{Euro}}}\right)$$

where *d*_between_ is synonymous *D*_xy_ between North American and European *D. pulex* and *p*_NAm_ and *p*_Euro_ are the levels of synonymous polymorphism within‐species. *D*_xy_ was calculated using *PopGenome v2.7.5* across all samples for each species (Revell 2019).

We performed demographic inference with *moments v1.1.0* (Jouganous et al. 2017) in *python3*. We tested two models: one with‐migration (‘*Split* + *Migration*’) and one without‐migration (‘*Split*’). For the former model, we used *moments*' *split_mig* model. For the latter, we used *split_mig* with the migration rate fixed at zero. We ran inference on 20 × 20 SFS projections until model convergence and classified a shared polymorphism as an allele whose allele frequency is above 1/20 in both species. Thus, the proportion of shared polymorphisms reported in this analysis is less than reported for the larger dataset (Table S2). We chose to use 20 × 20 SFS projections instead of the whole dataset because McCoy et al. (2014) showed that there is a marginal performance increase past even small projection sizes of 10 × 10. Also, we wanted to have equal projection sizes between‐species and increase computational performance for bootstrapping. We used a fixed mutation rate of *μ* = 5.69 × 10^−9^ (Lynch et al. 2017). We first performed *moments* inference and optimization across 1,000 bootstrap replicates for both the ‘*Split* + *Migration*’ model and the ‘*Split*’ model to derive optimized priors for parameters, including population sizes, split times and migration rates. Using these optimized priors, we generated 2D SFSs for North American and European *D. pulex* under each bootstrap model. We present the mean projections of the 2D SFS based on the results from 1,000 bootstrap replicates for each model. Mean standardized residuals were calculated from the allele counts for each element of the 2D SFS with the following formula: $\frac{\text{Empirical}-{\text{Model}}_{x}}{\sqrt{{\text{Model}}_{x}}}$, where Model_*x*_ is ‘*Split + Migration*’ or ‘*Split*’.

### 2.10 Balancing Selection Statistics

We used *BetaScan v1* (Siewert and Voight 2017) to calculate the *β* ^1^ statistic within‐species using the folded SFS. We used the *α*_*b*_ statistic to estimate the proportion of shared polymorphisms under balancing selection between‐species‐pairs from Soni, Vos, and Eyre‐Walker (2022), where

$$\alpha_{b}=1-\frac{\mathrm{Pol}\_\mathrm{Syn}\times \mathrm{SP}\_\mathrm{NS}}{\text{Poly}\_\mathrm{NS}\times \mathrm{SP}\_\mathrm{Syn}}$$

and *Poly_Syn* and *Poly_NS* are within‐species synonymous and non‐synonymous polymorphisms, respectively, and *SP_Syn* and *SP_NS* are shared polymorphisms between‐species at synonymous and non‐synonymous sites.

### 2.11 Phylogenetic Tree Test Using Pairwise Cophenetic Distances

We tested the local sequence genealogy for trans‐specificity versus convergent evolution (Koenig et al. 2019; Nunez et al. 2021). This test used trees built from 500 bps flanking high‐frequency non‐synonymous shared polymorphisms (MAF > 0.25) and calculated the median pairwise cophenetic distances (CPD) between samples (Cardona et al. 2013). We extracted haplotypes from a *WhatsHap v1.1* phased VCF (Martin, Ebert, and Marschall 2023) from 30 high‐coverage individuals for each species. We chose 30 samples to keep the sample size consistent across species whilst decreasing convergence time. We aligned the phased haplotypes (*n* = 60 per species) using *mafft v7.475* and built trees using *iqtree2* (1,000 bootstraps).

### 2.12 Light Exposure Experiments on *Daphnia* Activity

We developed a behavioural assay to collect activity data on 12 European *D. pulex* clones using a DAM Trikinetics monitor (TriKinetics Inc., Waltham, MA). In total, we measured activity for 216 individuals. Each well has an infrared light beam that when broken by a *Daphnia* will count as an activity event. We exposed individuals to white, blue and dark lighting conditions using blackout boxes mounted with LEDs (Erickson et al. 2020). Individuals were placed inside a plastic assay tube with media (ASTM; Standard 2007) whilst each monitor collected activity over a 12‐h period, sampling every 5‐seconds. We excluded measurements during the first hour to allow individuals to adjust to their new environment. For 95% of the 5‐s intervals, 0 or 1 beam‐breaks were recorded and 99.9% of intervals had four or fewer beam‐breaks. Therefore, for each interval, we converted the number of beam‐breaks recorded into a binary variable (≥ 1 beam‐break vs. 0 beam‐breaks) and calculated activity as the fraction of 5‐s intervals with more than one beam‐break per individual. We modelled activity with a generalized linear mixed effect model using *lme4 v1.1–27.1* (Bates et al. 2015) and performed likelihood ratio tests between the following models:

Model1:y∼Light+Clone+Block+ε

Model2:y∼Light+Genotype+Clone+Block+ε

Model3:y∼Light+Genotype+Light:Genotype+Clone+Block+ε

In these models, *y* is the fraction of intervals with activity, *Light* is the fixed effect of light treatment (white, blue and dark), *Genotype* is the fixed effect of genotype at the opsin (BLOP), *Light:Genotype* is the fixed interaction effect, (1|*Clone*) is the random effect of clone, (1|*Block*) is the random effect of one of the three experimental blocks run over successive weeks and ε is the binomial error with weights equal to the number of 5‐s intervals (ca. 7,800). We conducted likelihood ratio tests between each model using the *anova()* function in *R* (Table S3). In addition, we performed an analysis that models elapsed time in the experiment as a fixed effect and includes the individual *Daphnia* as a random effect to account for repeated measures. The results of that analysis are in line with the simple model presented here and are shown in Table S4.

### 2.13 BLOP Orthologs

We identified orthologs of the BLOP (*Daphnia11806* in the D84A annotation) by BLASTing the amino acid sequence against the NCBI database using *blastp v2.13.0* (Sayers et al. 2022).

### 2.14 Statistics and Visualization

Most analyses were performed using *R v3.6.2–4.0.3* (R Core Development Team 2013). We used the following packages for analysis and visualization: *tidyverse v1.3.1* (Wickham et al. 2019), *ggplot2 v3.3.5* (Villanueva and Chen 2019), *ggtree v2.0.4* (Xu et al. 2022), *ape v5.4‐1* (Paradis and Schliep 2019), *patchwork v1.0.1* (Thomas Lin Pedersen 2022), *data.table v1.12.8* (Dowle and Srinivasan 2023), *foreach v1.4.7*, *doMC v1.3.5* (Daniel et al. 2022) and *SeqArray v1.26.2* (Zheng et al. 2017). Principal component analysis (PCA) of SNPs was conducted in *SNPRelate v1.24.0* (Zheng et al. 2012). Linkage disequilibrium (*r* ^2^) between adjacent SNPs was calculated using *plink v1.9* (Purcell et al. 2007).

## 3 Results

### 3.1 Thousands of *Daphnia* Genomes

**FIGURE 1:**
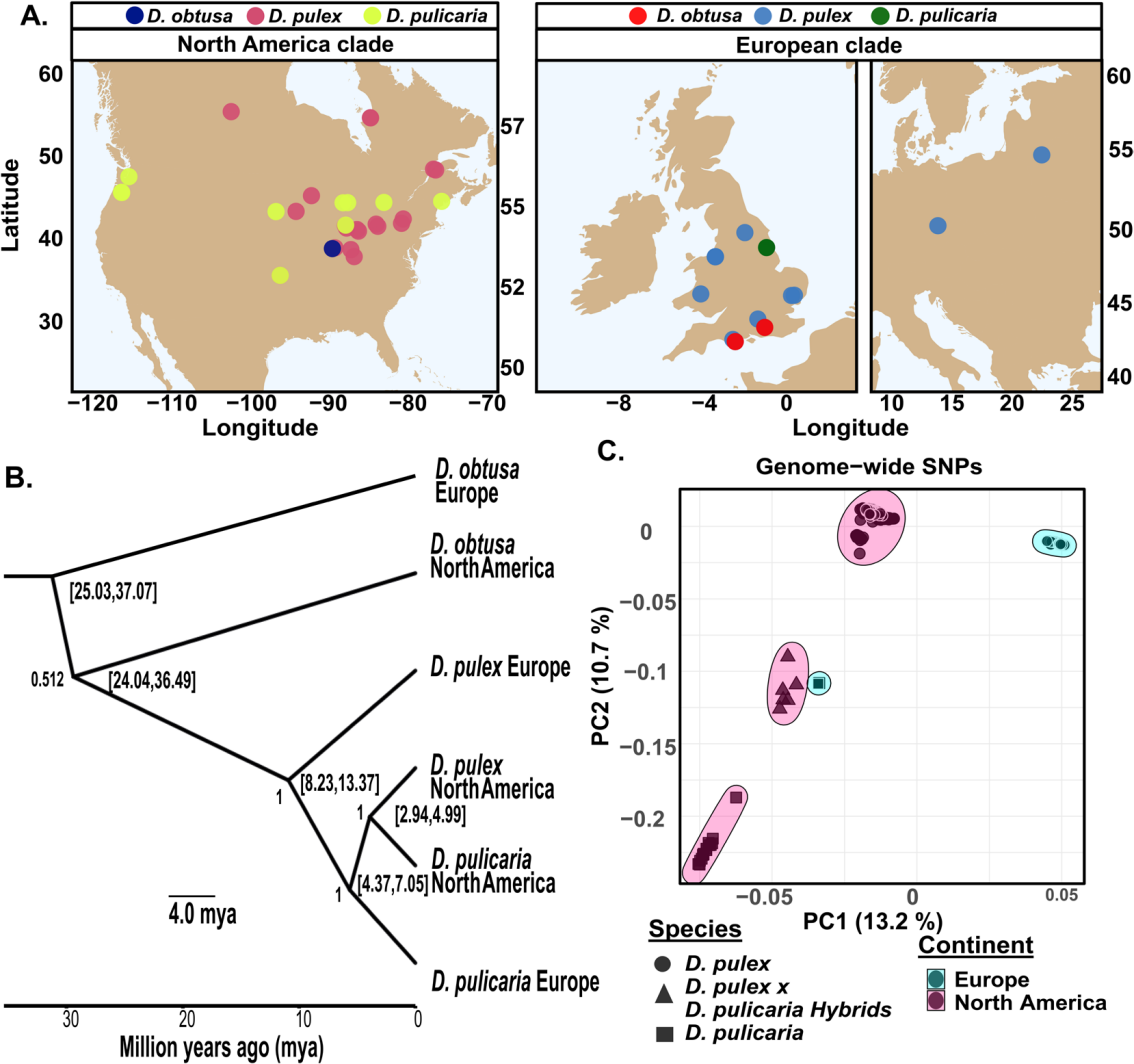
Genetic divergence of the *Daphnia pulex* species complex. (A) Sample origin of the North American and European clades, each consisting of *D. pulex* , *D. pulicaria* and *D. obtusa* . Most of the European clade has samples in the United Kingdom but there is one sample in both the Czech Republic and Lithuania as shown in the rightmost subfigure. (B) Time‐constrained phylogenetic tree restricted to two representative individuals within each species based on 3,000 BUSCO gene SNPs. This consensus tree is rooted with European *D. obtusa* to have 31 million years of divergence. Bracketed values are 95% confidence intervals in millions of years ago (mya). Node labels indicate the posterior probabilities estimated from one million bootstraps. (C) The principal component axes (PC1 and PC2) using filtered genome‐wide SNPs. The proportion of variation explained by each PC is shown in parentheses. We restricted the PCA to the *D. pulex* and *D. pulicaria* taxa because the *D. obtusa* taxa are so distantly related.

We first assembled short‐read genomic data for 2,321 samples of *D. pulex* , *D. pulicaria* and *D. obtusa* collected from North American and European ponds (Figure 1A). This includes whole genomes published elsewhere, along with known hybrids of North American *D. pulex* and North American *D. pulicaria* , and 93 European samples of *D. pulex, D. obtusa* and *D. pulicaria* reported here for the first time (see Table S1). The SNPs that we used represent within‐species SNPs, fixed differences, and shared polymorphisms between these taxa. The vast majority (97%) of the samples we used are either North American or European *D. pulex* , and therefore most of our analysis focuses on these taxa. Because lineages could be clonally derived from a recent common ancestor, each sample was assigned to a multi‐locus genotype using the filtered genome‐wide SNP set (MLG; Table S1). In all analyses, unless otherwise noted, we restricted to one sample per MLG (*N* = 1,173).

### 3.2 North American and European *D. pulex* are Distinct Species

To evaluate the nuclear phylogeny of the *D. pulex* species complex and to determine if North American *D. pulex* and European *D. pulex* are distinct taxa, we built a time‐constrained phylogenetic tree using BUSCO gene SNPs (Figure 1B). The tree omitted hybrids between North American *D. pulex* and *D. pulicaria* because they prevented model convergence. The nodes that split the *D. pulex* species complex are generally well‐supported. We estimate that the split‐time between North American and European *D. pulex* is around 10 million years ago (95% CI 8.23, 13.37; Figure 1B). Previous studies based on mitochondrial and nuclear markers also showed reciprocally monophyletic relationships between North American and European *D. pulex* (Vergilino et al. 2011; Marková et al. 2013). Similarly, our mitochondrial phylogeny supports this reciprocally monophyletic relationship between North American and European *D. pulex* . In contrast, the mitochondrial tree shows that North American *D. pulex* and North American *D. pulicaria* are not reciprocally monophyletic (Figure S2). The recent split‐time between North American *D. pulex* and *D. pulicaria* (Ye et al. 2022), their propensity to hybridize (Pantel, Juenger, and Leibold 2011) and discordant phylogenies support the hypothesis that North American taxa are in the process of incipient speciation (Heier and Dudycha 2009).

We performed PCA of *D. pulex* , *D. pulicaria* and known hybrids using a genome‐wide SNP dataset (Figure 1C). The first and second PC axes are significantly different between the North American and European *D. pulex* , *D. pulicaria* and hybrid species groups (ANOVA PC1: *F*_4,1154_ = 70,617, *p <* 2 × 10^−16^; ANOVA PC2: *F*_4,1154_ = 27,940, *p <* 2 × 10^−16^). Intriguingly, European *D. pulicaria* clusters near the known hybrids of North American *D. pulicaria* and *D. pulex* (Jackson et al. 2021; Tucker et al. 2013); below we test whether the samples identified as *D. pulicaria* collected in Europe are related hybrids between North American taxa or are themselves hybrids.

North American and European *D. pulex* possess marked differences in levels of diversity, consistent with long‐term divergence. Principal component clusters are more dispersed amongst North American *D. pulex* than they are amongst European *D. pulex* , suggesting higher genetic variability within the North American clade (Figure 1C). Demographic history reconstruction further highlights these distinctions: the effective population size (*N*_e_) for North American *D. pulex* is estimated at approximately 500,000 (95% CI: 487,787, 522,128) over a timescale of 1 kya to 1 mya, compared to ~200,000 (95% CI: 225,775, 235,111) for European *D. pulex* when using *MSMC2* (Figure S3). Estimates of historical population size from *MSMC2* do not incorporate the potential for contribution from the resting egg bank. To account for the contribution from the egg bank, and to estimate the egg bank deposition rate (the inverse of the per‐generation germination rate, *β*) we ran *eSMC* with either 5 or 10 generations of parthenogenesis between every sexual generation (Figure S3). *eSMC* estimates of *N*_*e*_ are similar in scale to one other when assuming 5 generations per year to the *MSMC2* results: North American *D. pulex* (95% CI: 42,600, 43,211) and European *D. pulex* (95% CI: 26,574, 27,039). Under 10 generations per year, the estimates of *N*_*e*_ are increased: North American *D. pulex* (95% CI: 149,228, 152,899) and European *D. pulex* (95% CI: 123,245, 127,387). The *eSMC* analysis also suggests both species have substantial contributions to the egg bank because they have moderate rates of hatching each generation: *β*_Euro_ = 0.336 (95% CI: 0.332, 0.34) and *β*_NAm_ = 0.322 (95% CI: 0.321, 0.323) under an assumption of 5 generations per year. Doubling the generations to 10 per year increases *β*, suggesting a hatching rate of *β*_Euro_ = 0.69 (95% CI: 0.68, 0.71) and *β*_NAm_ = 0.65 (95% CI: 0.644, 0.65). Taken together, our analysis of the genomes supports the conclusion that *D. pulex* found in North America and Europe are different species that likely diverged millions of years ago.

### 3.3 Hybridization in the *D. pulex* Species Group

**FIGURE 2:**
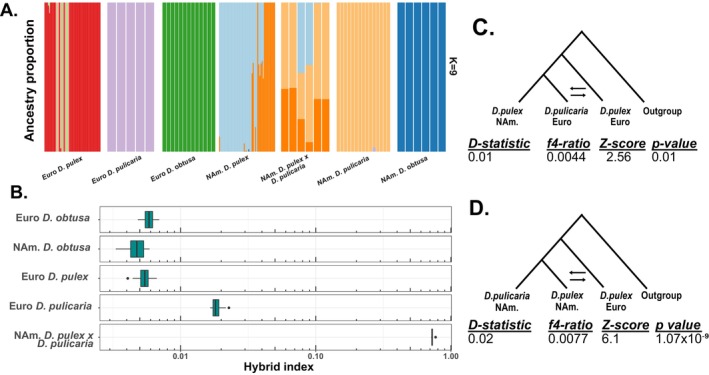
Hybridization across the *D. pulex* species complex. (A) *ADMIXTURE* plot of the *D. pulex* species complex with *k* = 9 having the minimal cross‐validation error. Each color represents a unique ancestry group for each sample. (B) We identified fixed differences between North American *D. pulex* and *D. pulicaria* and calculated the hybrid index (i.e., the proportion of fixed differences between North American *D. pulex* and *D. pulicaria* that are heterozygous in any individual of the tested species) in a randomly chosen individual from the remaining taxa. The boxplot shows the distribution of the hybrid index from randomly sampled clones (one per MLG). (C, D) Introgressions tests using various four‐species trees. The outgroup is European *D. obtusa* in all tests. *D‐statistic* and *f4‐ratio* describe the extent of introgression between the 2nd and 3rd taxa on the tree being tested.

Hybridization is common amongst North American *D. pulicaria* and *D. pulex* species (Pantel, Juenger, and Leibold 2011; Vergilino et al. 2011; Marková et al. 2013), however, signals of hybridization between North American and European *Daphnia* remain less well understood. Hybridization between European *D. pulicaria* and North American or closely related circumarctic species is not recent or is with other members of the complex North American *D. pulex‐pulicaria* sub‐group. For example, an *ADMIXTURE* analysis reveals that European *D. pulicaria* has distinct clusters from other species, whilst the recent North American *D. pulex‐pulicaria* hybrid displays split ancestry between North American *D. pulex* and *D. pulicaria* (Figure 2A; Alexander and Lange 2011). We also examined heterozygosity at fixed differences (i.e., hybrid index) between North American *D. pulex* and North American *D. pulicaria* in European *D. pulicaria* and North American *D. pulex* ‐*pulicaria* hybrids. These fixed differences are heterozygotes 70% of the time in North American hybrids, but only 2% of the time in European *D. pulicaria* , suggesting a distinct evolutionary history of the European *D. pulicaria* clade (Figure 2B). In summary, our findings imply that European *D. pulicaria* is likely a member of the speciose North American *D. pulex* species sub‐group, consistent with previous reports of a circumarctic *D. pulex* lineage predominant across Northern Eurasia (Colbourne et al. 1998).

Signals of hybridization are weak between European *D. pulex* and other taxa. For instance, signals of admixture between European *D. pulex* and European *D. pulicaria* are weak (*D* = 0.01, *f4‐ratio* = 0.0044, *p* = 0.01; Figure 2C) as are signals of admixture between European *D. pulex* and North American *D. pulex* (*D* = 0.02, *f4‐ratio* = 0.0077, *p* = 1.07 × 10^−9^; Figure 2D). *ADMIXTURE* analysis suggests that European *D. pulex* forms several distinct ancestry groups that do not appear within any species of North American *Daphnia* (Figure 2A). Only ~0.5% of fixed differences between North American *D. pulex* and North American *D. pulicaria* segregate as heterozygous sites in European *D. pulex* (Figure 2B). These results suggest that European *D. pulex* are distinct from the remaining taxa and likely do not have a recent history of hybridization with the other species studied.

### 3.4 Extent of Shared Polymorphism Between North American and European *D. pulex* is Not Explained by Incomplete Lineage Sorting or Recent Migration

For species with deep split‐times and low levels of migration or hybridization, we expect few shared polymorphisms to exist if such polymorphisms are neutral. For instance, based on a simple neutral model with no migration (Novikova et al. 2016; see Section 2) we expect to observe 336 shared polymorphisms at synonymous sites given the split‐time between North American and European *D. pulex* . Yet, we observe at least 11,000 shared synonymous SNPs between these species (Table S2).

**FIGURE 3:**
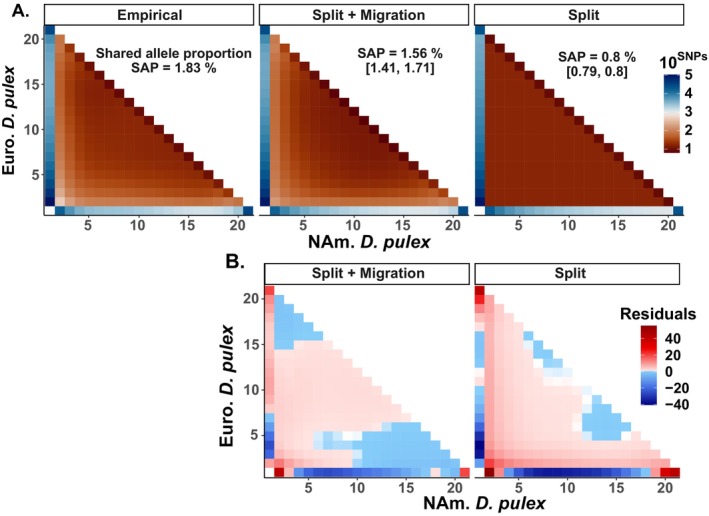
An excess of shared polymorphisms between North American and European *D. pulex* . (A) Demographic model inference between North American and European *D. pulex* based on the folded site‐frequency spectrum (SFS). The empirical SFS is constructed from the genome‐wide SNP dataset. The split with migration (‘*Split + Migration*’) and split without migration (‘*Split*’) models were generated from *moments* and we are showing the mean projection based on 1,000 bootstraps. The x‐ and y‐axis use a 20 × 20 SFS projection. (B) Average standardized residuals for both models tested against the empirical SFS.

This prediction does not account for historic migration, so we performed demographic inference on the two‐dimensional site‐frequency spectrum (2D SFS) using *moments* (Figure 3A; Jouganous et al. 2017). First, we contrasted two models, one that allows constant migration (*Split + Migration*) and one where the migration rate was set to zero after population divergence (*Split*). The ‘*Split + Migration*’ model is the best model based on the mean Bayesian information criteria (BIC; ‘*Split + Migration*’ BIC = 20,637 and ‘*Split*’ BIC = 33,216). Notably, the ‘*Split*’ model severely underpredicts the number of shared polymorphisms, reflecting that incomplete lineage sorting alone is insufficient to explain the abundance of shared SNPs. The ‘*Split + Migration*’ model itself underpredicts the number of shared polymorphisms by 25% (Figure 3A), and the model prediction shows a notable deficit of common shared SNPs and an excess of shared SNPs that are at low frequencies compared to the empirical SFS (Figure 3B).

### 3.5 Selective Forces Acting on Shared Polymorphisms

**FIGURE 4:**
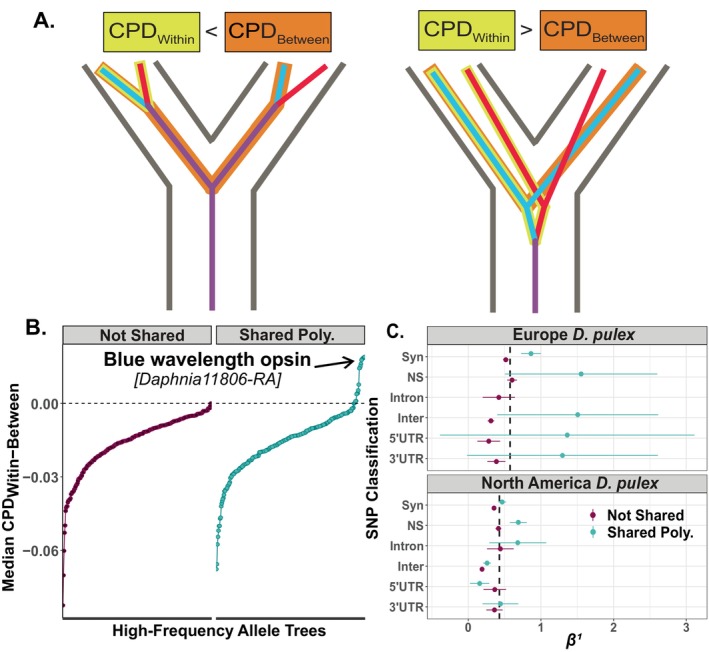
Convergent evolution, trans‐specificity and signatures of balancing selection. (A) Visualization of two adaptive hypotheses that produce shared polymorphisms, convergent evolution on the left and trans‐specificity on the right. For each tree, we calculated the median pairwise cophenetic distance as the distance within‐species (CPD_Within_; yellow highlighted pair) – between‐species (CPD_Between_; orange highlight) for shared polymorphisms and non‐shared polymorphisms. CPD_Within‐Between_ < 0 describes the consensus species‐tree topology (Left), whilst CPD_Within‐Between_ > 0 describes an allele‐specific tree topology consistent with multiple linked trans‐specific polymorphisms (Right). The red and blue branches indicate examples of shared polymorphisms between‐species. (B) CPD_Within‐Between_ for non‐synonymous shared SNPs and non‐shared SNPs above 0.25 minor allele frequency (MAF) in both species. Each allele‐tree was made from 30 samples from North American and European *D. pulex* . At the focal SNP, we extracted 500 bps surrounding the focal SNP. (C) *β* ^1^ is a statistic that detects balancing selection. We show the mean with 95% standard errors for several SNP classifications (SYN = synonymous, NS = non‐synonymous, Intron = intronic, Inter = intergenic, ±500 bps upstream and downstream variants, 5′ UTR = 5′ untranslated region, 3′ UTR = 3′ untranslated region). The dotted vertical line is the average *β* ^1^ within each species.

European and North American *D. pulex* share more polymorphisms than expected by neutral, demographic models, suggesting selection may maintain these polymorphisms. We aimed to differentiate old, trans‐specific polymorphisms versus convergently evolved polymorphisms by constructing phylogenetic trees for genes with shared polymorphisms. If convergent evolution occurred, gene‐trees would match the species‐tree, with haplotypes from the same species clustering together (Figure 4). However, in genes with old trans‐specific polymorphisms, gene‐trees might cluster by allele type, not species. However, for this to occur there must be multiple trans‐specific polymorphisms in close linkage as a single trans‐specific polymorphism would not be sufficient for the gene‐tree to differ from the species tree (Gao, Przeworski, and Sella 2015). Our analysis therefore can highlight genes with multiple linked trans‐specific polymorphisms, which may be targets of long‐term balancing selection.

We summarized the topology of gene‐trees by calculating the pairwise CPD (Cardona et al. 2013) within and between haplotypes of the same‐species from gene‐trees that contain high‐frequency (MAF > 0.25), non‐synonymous, shared polymorphisms. When gene‐trees resemble the species‐tree topology, the within‐species distances will be lower than the between‐species distances (CPD_Within_ − CPD_Between_ = CPD_Within‐Between_ < 0; Figure 4A). However, if alleles from two species cluster together, and are discordant with the species‐tree, the within‐species distances will be larger than between‐species distances (CPD_Within‐Between_ > 0). A small number of gene‐trees surrounding shared polymorphisms have a positive CPD_Within‐Between_ value, consistent with old trans‐specific haplotypes (Figure 4A). However, most shared polymorphisms have negative CPD_Within‐Between_ values (Figure 4B), consistent either with convergent evolution or trans‐specificity.

Regardless of whether shared polymorphisms arose via convergent evolution, or prior to the species‐split, they could have been subject to balancing selection. To test this hypothesis, we first calculated *α*_*b*_, a statistic to estimate the proportion of non‐synonymous sites under balancing selection using counts of species‐specific alleles and shared polymorphisms (Soni, Vos, and Eyre‐Walker 2022). We found that *α*_*b*_ is significantly positive across the genome, indicating that balancing selection is influencing non‐synonymous shared polymorphisms (*α*_*b*_ = 0.082 [0.05, 0.114], *p* = 1.5 × 10^−6^). Next, we calculated *β* ^1^, an SFS‐based statistic for detecting signals of balancing selection (Siewert and Voight 2017) at both shared and control SNPs. We found that *β* ^1^ at shared polymorphisms is significantly larger than zero in both species for non‐synonymous SNPs (one sample *t*‐test: Euro. *t* = 7.8, *df* = 270, *p* = 1.75 × 10^−13^; NAm. *t* = 18.8, *df* = 1,563, *p* = 2.2 × 10^−16^; Figure 4C). Shared synonymous sites also show significantly elevated *β* ^1^ in both species (NAm. *t* = 25.12, *df* = 4,367, *p* = 2.2 × 10^−16^; Euro. *t* = 20.41, *df* = 1,481, *p* = 2.2 × 10^−16^; Figure 4C).

### 3.6 Trans‐Specific Polymorphisms at an Opsin Affects Behavior and Shows Evidence of Genetic Overdominance

**FIGURE 5:**
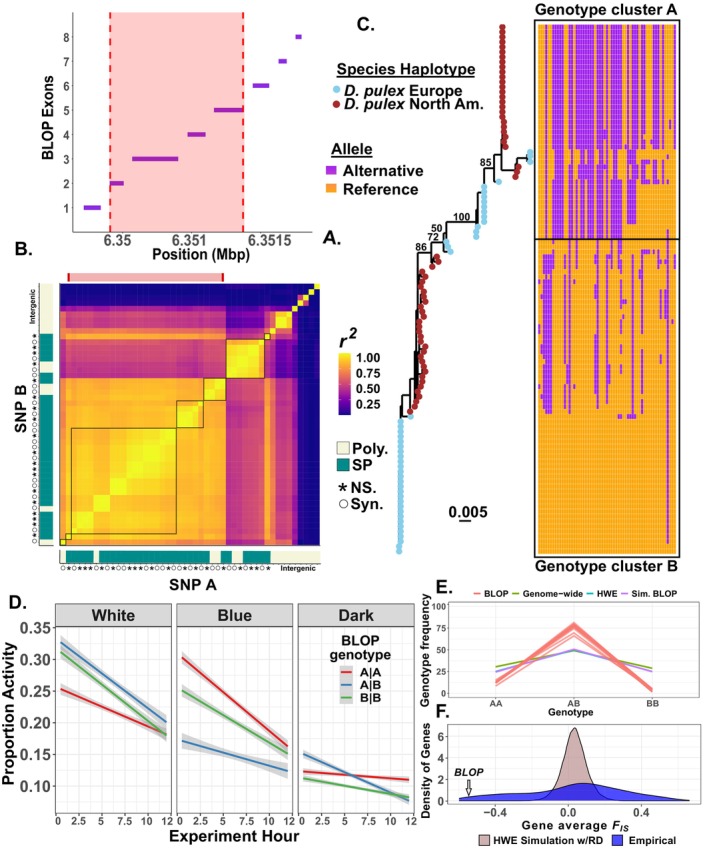
Behavioral and fitness effects of trans‐specific SNPs at a blue wavelength opsin. (A) Gene structure showing the length and position of exons within BLOP (*Daphnia11806*). The shaded red region indicates the location of a large high‐linkage block identified in panel B. (B) Pairwise linkage disequilibrium (*r* ^2^) for every SNP within BLOP for European *D. pulex* , filtered for SNPs with a MAF > 0.01. The right and bottom tile objects indicate whether the SNP is polymorphic (Poly; khaki) or a shared polymorphism (SP; blue‐green). NS refers to non‐synonymous polymorphism and Syn refers to synonymous polymorphism represented by asterisks and open circles respectively. The grey boxes indicate within‐exon SNP comparisons, exon 8 did not have any relevant SNPs. (C) Allele‐tree made from the gene for a subset of phased samples of North American and European *D. pulex* . Tip symbols indicate whether the samples are North American or European *D. pulex* . Numbers indicate bootstrap support. The included haplotype plot and multiple‐sequence alignment showcase the presence of each SNP within the gene, colored for whether the allele is derived (purple) or reference (gold). (D) The activity of individual European *D. pulex* was measured for 12 h for three genotypes in three different light conditions. Lines represent the best fit and 95% standard errors. (E) Average frequency of F1 genotypes expected based on a double heterozygous cross (i.e., AB × AB) using empirical read depth at each SNP. ‘BLOP’ is the empirical segregation of trans‐specific polymorphisms within the BLOP gene ‐ amongst F1 genotypes. ‘Genome‐wide’ is the segregation for SNPs based on the read depth. ‘HWE’ is the segregation pattern expected for Hardy Weinberg equilibrium. ‘Sim. BLOP’ is the segregation pattern expected for the SNPs within BLOP based on empirical read depth. (F) Distribution of average gene *F*_IS_. ‘HWE Simulation w/RD’ is the expected *F*_IS_ for each gene based on the empirical read depth for each SNP within every gene and ‘Empirical’ is the average *F*_IS_ across genes. The small arrow denotes where the gene average for the blue wavelength opsin falls along the empirical distribution.

Of the common, non‐synonymous shared polymorphisms, 14 (5%) have positive CPD_Within‐Between_ values (Figure 4B). Almost all of these shared polymorphisms (13/14) are within a rhabdomeric blue wavelength opsin (BLOP) gene (Brandon, Greenwold, and Dudycha 2017). BLOP is found as a single‐copy in European and North American *D. pulex* (Figure S4A). The 13 non‐synonymous shared SNPs reside across several exons (Figure 5A) and encompass a large linkage block within European *D. pulex* (*r* ^2^ > 0.7 across ~1.5 kbps; Figure 5B,C), thereby explaining the gene‐tree species‐tree discordance (Figures 4B and 5C) and suggest that these alleles are trans‐specific polymorphisms that predate the split between North American and European *D. pulex* .

If these haplotypes at the BLOP have been maintained prior to the split between North American and European *D. pulex* 10 mya (Figure 1C), they may have a functional effect. To test this hypothesis, we measured the light‐induced activity of European *D. pulex* clones that harbour distinct haplotypes bearing alternate shared alleles. We first assigned clonal haplotypes to one of two genetic clusters (Figure 5C) and tested the activity levels of all three genotypes (AA, AB and BB) in different light conditions. We found that genotype has a significant effect on activity that is dependent on light conditions (*χ* ^2^ = 5,849.71, *df* = 4, *p* < 2 × 10^−16^; Table S3). In general, all genotypes had low activity in dark conditions. Heterozygotes have the highest activity levels when exposed to white light yet have the lowest activity when exposed to blue light consistent with shifts between genetic overdominance and underdominance affecting behavior (Figure 5D).

Overdominance affecting behavior could also translate into overdominance affecting fitness. If trans‐specific polymorphisms at the BLOP cause overdominance in fitness, heterozygotes should be more common than expected under Hardy–Weinberg equilibrium (HWE). To test the hypothesis, we examined segregation patterns of trans‐specific SNPs at the BLOP amongst F1 offspring derived from a cross between two clones that are both heterozygous for the trans‐specific haplotypes we identified. These clones were previously referred to as ‘super‐clone A’ and ‘super‐clone C’ by Barnard‐Kubow et al. (2022). Both clones had reached high frequency in the southern English (Dorset) pond D8 by the end of the 2017 growing season. In 2018, most individuals in the D8 pond were the F1 offspring between super‐clone A and C (74%) enabling us to directly test if there is an excess of heterozygotes relative to the expected Mendelian segregation patterns amongst the F1s. First, we calculated the frequency of AA, AB and BB genotypes at the BLOP locus compared to the rest of the genome, without downsampling to one clone per MLG. In this way, we are able to see how all samples collected were affected by selection at the BLOP locus. We find that there is an excess of heterozygotes in the wild‐caught individuals compared to expectations from HWE and compared to random SNPs in the genome or other trans‐specific polymorphisms (Figure 5E). We calculated the distribution of *F*_IS_, a measure of the departure of HWE, at genes across the genome and found that the BLOP gene is amongst the most strongly negative *F*_IS_ compared to other genes (*F*_IS_ = −0.54; Figure 5E). The BLOP gene has amongst the smallest 2.6% of *F*_IS_ values. Even if we examine the genotype distribution by only sampling one individual per MLG, we still observe an excess of heterozygotes (Figure S5C–F). Finally, we examined genotype frequencies in lab‐generated A × C and C × C F1s. In contrast to our field‐sampled individuals, we do not observe an excess of heterozygotes from a lab‐generated cross of the same clones (Figure S5A–E).

## 4 Discussion

In this study, we examined the evolutionary forces that generate and maintain shared polymorphisms between two taxa in the *D. pulex* species complex. We used whole‐genome sequences coupled with polymorphism data to resolve the nuclear phylogeny of members of this species group and to evaluate mechanisms that can generate shared polymorphisms between‐species. We show that there is an excess of shared polymorphisms between North American and European *D. pulex* that cannot be explained by neutral or demographic processes, thereby implicating some form of natural selection as a force maintaining polymorphism. For one gene, a blue wavelength opsin, we show that shared polymorphisms are ancient, predating speciation and has functional consequences on behavior and fitness in the wild.

### 4.1 Phylogenetics of the *D. pulex* Species Group

Members of the genus *Daphnia*, and the *D. pulex* species group in particular, have proven challenging from a taxonomic perspective since their early description. For instance, Leydig separated *D. pulex* from *D. magna* and *D. longispina* (Leydig 1860, p. 117), but did not further describe divisions. Richard (1896), identified *D. obtusa* as a distinct species from *D. pulex* (p. 260) but also described ten subspecies of *D. pulex* found across the Americas and Eurasia (p. 232–255). Scourfield (1942), reinforced the view that *D. obtusa* and *D. pulex* are distinct species and emphasized that this species group represents several lineages in various stages of speciation. Johnson (1951), in his description of British members of the *D. pulex* group, noted that American forms resembling species in the *D. pulex* group are not likely monophyletic with Eurasian species of the same name, although these taxanomic naming conventions have persisted (e.g., Brooks 1957b; Omilian and Lynch 2009; Ye, Pfrender, and Lynch 2023). The challenge of morphological classification in the *D. pulex* group stems from a limited number of diagnostic characteristics (Brooks 1957b; Dodson 1981), coupled with phenotypic plasticity (Colbourne et al. 1997), intraspecific mating type variation (Heier and Dudycha 2009; Jose and Dufresne 2010) and cytological variation (Gómez et al. 2016; Hosseinie 1966). However, recent phylogenetic analysis of mitochondrial markers has shown the *D. pulex* group consists of many distinct lineages and that the deepest splits within the *D. pulex* species group occur between Eurasian and North American taxa (Crease et al. 2012; Ye et al. 2022). Consistent with these results, allopatric speciation has been estimated to account for roughly 40% of cladogenetic events within *Daphnia* (Adamowicz et al. 2009), a process possibly enhanced by cycles of glaciation (Chin and Cristescu 2021). We show that substantial genetic division exists between North American and European taxa and that these taxa are separated by millions of years (Figure 1). Given the deep split time between members of the *D. pulex* species group, it is likely that they have distinct features ranging from their response to environmental stimuli to their impact on the ecosystem. Further study of the behavioral, physiological and ecological interactions that distinguish these taxa is warranted.

The complicated nature of the *D. pulex* species group is compounded by incomplete reproductive isolation. North American *D. pulex* and North American *D. pulicaria* are known to hybridize in the wild (Xu et al. 2015; Ye et al. 2019). Hybrids between these lineages are obligately asexual and fail to produce functional males (Tucker et al. 2013; Xu et al. 2015; Ye et al. 2019). These post‐zygotic reproductive incompatibilities are a hallmark of taxa undergoing incipient speciation (Coughlan and Matute 2020). Consistent with this view, we show that the split‐time between North American *D. pulex* and *D. pulicaria* based on the nuclear genome is recent, within 3 mya (Figure 1B). Our estimate is consistent with a study made from mitochondrial genomes (Colbourne et al. 1998; Marková et al. 2013), but older than another using a limited number of nuclear markers (Omilian and Lynch 2009). Nonetheless, genomic data clearly show that hybridization between these North American lineages occurs (Xu et al. 2015; Ye et al. 2019; Figure 2). Previous analysis of mitochondrial markers placed European *D. pulicaria* as a sister to the North American *D. pulex/pulicaria* clade (Marková et al. 2013), a result consistent with the nuclear phylogeny we constructed (Figure 1B). European *D. pulicaria* also shows evidence of hybridization with members of the North American *D. pulex/pulicaria* clade (Figure 2B), although such hybridization is not likely recent or could have occurred with other lineages in this complex that are not represented here. Although the North American taxa, along with European *D. pulicaria* show signals of hybridization with each other, European *D. pulex* appears to be a well‐defined species. We show that European *D. pulex* split from the other *D. pulex/pulicaria* taxa approximately 10 mya (Figure 1B) and has little to no evidence of recent hybridization (Figure 2B–D). More extensive sampling of the *D. pulex* species group across the Northern hemisphere will be necessary to more exhaustively test complex patterns of hybridization and introgression.

### 4.2 The Evolution of Shared Polymorphisms

Polymorphisms that are shared between‐species represent a particularly interesting class of mutations because they can reflect a wide variety of evolutionary processes. On the one hand, shared polymorphisms could reflect neutral processes when they occur between closely related species. For example, species that diverged relatively recently will share many neutral polymorphisms because there has been insufficient time for drift to cause fixation or loss (Hobolth et al. 2011), and because of ongoing gene‐flow (Payseur and Rieseberg 2016). Whilst the presence of neutral shared polymorphisms due to incomplete lineage sorting or gene‐flow is important for understanding features such as historical population size (Suh, Smeds, and Ellegren 2015) or barriers to migration (Kutschera et al. 2014), they can obscure selective forces such as convergent adaptive evolution or balancing selection that can also generate or maintain shared polymorphism. Therefore, to examine these selective forces, it is important to identify species that have diverged long enough ago that incomplete lineage sorting and ongoing gene‐flow are limited. Our work identifies European and North American *D. pulex* as two such species because of their relatively deep split‐time and limited evidence for hybridization.

We show that there are tens of thousands of polymorphisms that are shared between European and North American *D. pulex* (Figure 3A, Table S2) and suggest that natural selection is responsible for their presence. Natural selection has often been implicated as playing a key role in maintaining shared polymorphism. For instance, immune‐related genes of invertebrates (Cornetti et al. 2024), vertebrates (Aguilar et al. 2004; Azevedo et al. 2015; Klein et al. 1993) and plants (Klein et al. 1998; Sutherland, Tobutt, and Robbins 2008; Novikova et al. 2016) are routinely found to possess trans‐specific polymorphisms that are older than the species split and are thought to be maintained as polymorphic via mechanisms such as negative frequency dependence or genetic overdominance (Key et al. 2014). In other cases, shared polymorphisms in a variety of taxa have possibly arisen via convergent evolution to common selective pressures such as pathogens (Těšický and Vinkler 2015) and have been subject to balancing selection in both species (Solberg et al. 2008).

The shared non‐synonymous polymorphisms that we identified have gene‐trees that largely reflect the species‐tree (Figure 4). Taken at face value, this result is consistent with convergent evolution. Others have suggested that widespread convergent evolution is an unlikely mechanism generating shared polymorphisms (Klein et al. 1993). Is this conclusion valid for *Daphnia*? The probability of a beneficial mutation arising in a population is a function of its census size (Pennings and Hermisson 2006) and its establishment in a population is a function of the selective value of the mutation (Haldane 1927). Whilst the long‐term effective population size of both European and North American *D. pulex* is somewhat limited (*N*_e_ < 1 million; Figure S3), the census size at any single pond or lake can be quite large, possibly reaching into the millions of individuals (Dudycha 2004), whilst the global census size of either species can reach upwards of 10^12^ individuals (Buffalo 2021). Therefore, across the species range, these taxa are not likely mutation‐limited. Recurrent *de novo* evolution of beneficial mutations has been hypothesized to occur rapidly and contribute to within‐population variation in male production rates (Barnard‐Kubow et al. 2022) and morphological responses to predators (Becker et al. 2022). Positive and fluctuating selection have been inferred to drive adaptive differentiation amongst and within *Daphnia* populations (e.g., Barnard‐Kubow et al. 2022; De Meester, Boersma, and Spaak 1999; Reger et al. 2018; Yampolsky, Schaer, and Ebert 2014) and balancing selection has been inferred to maintain heterozygosity within‐populations, suggesting that selection on new beneficial mutations could be strong enough to prevent beneficial mutations from being lost (Chaturvedi et al. 2021; Flynn et al. 2017; Lynch 1987; Lynch et al. 2023). Taken together, it is conceivable that such shared polymorphisms between North American and European *D. pulex* arose independently. On the other hand, it is possible that many of these shared polymorphisms are old and predate speciation, even if the gene‐trees surrounding the shared polymorphism are consistent with the species‐tree. For instance, if a SNP is a trans‐species polymorphism and is old, then the genetic footprint surrounding it will become eroded due to recombination (Gao, Przeworski, and Sella 2015) and the signature of trans‐specificity will be lost. Regardless, the shared polymorphisms that we observe show signatures of balancing selection compared to non‐shared polymorphisms in the genome (Figure 4C), suggesting that even if the shared mutations arose via convergent evolution many are relatively old. Moreover, theoretical work has shown that balanced polymorphisms in facultatively sexual organisms have especially deep coalescent times, compared to obligately sexual organisms (Agrawal and Hartfield 2016), consistent with the signatures of elevated polymorphism that we observe using the *β* ^1^ statistic. Furthermore, the ratio of non‐synonymous to synonymous polymorphisms is higher for shared polymorphisms (0.58) than non‐shared polymorphisms in both North American and European *D. pulex* (0.53 and 0.46, respectively; Table S2). Therefore, it is likely that many shared polymorphisms are functional and subject to balancing selection.

### 4.3 Natural Selection Maintains Functional Trans‐Specific Polymorphisms in a Blue Wavelength Opsin Gene

We show that one gene, a blue wavelength opsin harbours trans‐specific mutations that predate the split between North American and European *D. pulex* (Figures 4B and 5C). At this locus, the gene‐tree differs from the species‐tree, a signal that is consistent with trans‐specific polymorphism (Charlesworth 2006; Fijarczyk and Babik 2015). This BLOP gene has 15 non‐synonymous trans‐specific polymorphisms, extensive heterozygosity (Figure S4B) and elevated linkage disequilibrium making it a high‐priority candidate for functional characterization.

Research into the North American *D. pulex* genome has shown ancient expansion of opsin genes that occurred over 145 mya (Brandon, Greenwold, and Dudycha 2017). Recent work showcases distinct selective pressures between North American *D. pulex* and *D. pulicaria* at opsins highlighting the complex patterns of selection acting upon them (Ye, Pfrender, and Lynch 2023). It could be that this blue wavelength opsin mediates behavioral responses like predator avoidance or vertical diel migration (Li et al. 2022). Our laboratory experimental work shows that alternate genotypes at the BLOP have variable behavioral activity patterns in response to different light conditions (Figure 5D). Indeed, it even appears that there are changes in dominance as a function of light treatment, a feature that is consistent with the long‐term persistence of balanced polymorphisms (Wittmann et al. 2017).

Our observations of a natural population in Dorset, England identified a fitness advantage of heterozygotes in the wild (Figure 5E), consistent with previous work in *Daphnia* (Haag and Ebert 2007; Hebert, Ferrari, and Crease 1982). Our result relies on temporal sampling of a single wild population along with the reconstruction of the pedigree using genomic data of individuals (Barnard‐Kubow et al. 2022). Barnard‐Kubow et al. (2022), show that two clones became dominant in a pond by the end of 2017 and then crossed with each other, producing a population of F1 offspring the following year. The two dominant clones were heterozygous for the trans‐specific polymorphisms at the BLOP and thus we expect their offspring to follow a simple Mendelian 1:2:1 ratio. In contrast, we observe an excess of heterozygous individuals at 81% (35/43 isofemale clones). This pattern is largely explained by heterozygous clones reaching higher frequency in the population by the time they were sampled, suggesting that heterozygotes had higher fitness and thus were more likely to survive. By contrasting genotype frequencies in F1s from the field and the lab (Figure S5), we conclude that the excess of heterozygotes in the field is not likely due to factors such as inbreeding depression or associative overdominance (Ohta 1971). Instead, the excess of heterozygotes in the field likely emerged due to the action of natural selection. Given the strong link between looming stimulus, movement and predator avoidance in *Daphnia* (Pijanowska and Kowalczewski 1997; Ringelberg 1999; Van Gool and Ringelberg 2003), we hypothesize that trans‐specific polymorphisms at the BLOP locus may play a role in conferring a fitness advantage by reducing encounters with predators or by facilitating migration through the water column.

## 5 Conclusion

Our study elucidates the evolutionary history and genetic structure of the *D. pulex* species complex and provides evidence that shared polymorphisms are common between North American and European *D. pulex* . We show that balancing selection broadly influences shared polymorphisms and that a small fraction predates the species‐split. We demonstrate the functional significance of shared polymorphisms across specific ecological contexts and show that polymorphisms at a blue wavelength opsin gene are associated with fitness in the wild. Whilst we present four hypotheses related to the origin and maintenance of shared polymorphism (hybridization, incomplete lineage sorting, convergence and balancing selection), these hypotheses are not mutually exclusive. Additionally, the evolutionary mechanisms presented as hypotheses will all be affected by background levels of recombination, historic shifts in *N*_e_ and patterns of positive and purifying selection acting upon the genome (Charlesworth 2009, 2006). Despite this challenge, we laid the groundwork for understanding the mechanisms by which genetic diversity is maintained between members of the *D. pulex* species group.

## Author Contributions

**Connor S. Murray:** conceptualization, data curation, formal analysis, investigation, methodology, project administration, software, visualization, writing – original draft, writing – review and editing. **Madison Karram:** methodology, investigation, writing – review and editing. **David J. Bass:** formal analysis, software, visualization, writing – review and editing. **Madison Doceti:** investigation, writing – review and editing. **Dörthe Becker:** investigation, resources, writing – review and editing. **Joaquin C. B. Nunez:** formal analysis, software, methodology, writing – review and editing. **Aakrosh Ratan:** formal analysis, software, writing – review and editing. **Alan O. Bergland:** conceptualization, formal analysis, funding acquisition, investigation, methodology, project administration, software, supervision, validation, visualization, writing – review and editing.

## Conflicts of Interest

The authors declare no conflicts of interest.

## Supporting information


Figures S1–S5.

Tables S1–S4.

## Data Availability

The D84A mitochondrial genome was uploaded to NCBI under accession number JAHCQT000000000. The 93 new genomes described here are available on NCBI under the accession: PRJNA982532. The metadata for samples are in Table S1. The VCF and GDS are deposited on dryad: https://doi.org/10.5061/dryad.wwpzgmsv9. Scripts and data are deposited on GitHub: https://github.com/connor122721/SharedPolymorphismsDaphnia.
