# Supplementary material for: Trans‐Specific Polymorphisms Between Cryptic Daphnia Species Affect Fitness and Behavior
Source: Mol Ecol. 2024 Dec 24;34(3):e17632. doi: 10.1111/mec.17632 (PMC11754708; doi:10.1111/mec.17632)
Supplement: Supplementary file 1 — Figures S1–S5. [file MEC-34-e17632-s001.docx]

**Supplemental Figure 1. No evidence of reference allele bias across the *Daphnia pulex* species complex. A)** Alluvial plot of the SNP classifications between assemblies of the European *D. pulex* (D84A) and the North American *D. pulex* (KAP4). **B)** Proportion of SNP classification changes when mapping to KAP4 exclusively. **C)** The number of classified SNPs that are exclusive to each assembly. **D)** For each species, we extracted representative individuals (ranging from N=5-100 depending on the number of samples per species) and 1,000 biallelic heterozygous BUSCO gene SNPs (100 bootstraps) to gauge the severity of reference allele bias across the genome. We calculated the proportion of the alternative and reference dosage within a given individual for each site. The x-axis measures the proportion of alternative to reference dosage for each SNP and we show the 95% quantiles and median.

**Supplemental Figure 2. Mitochondrial protein-coding tree for the *D. pulex* species complex.** A maximum-likelihood tree with the “TN+F+I+G4” model output from IQTree2. The tree is rooted with *D. magna* as an outgroup. Bootstrap supports are listed as node labels.

**Supplemental Figure 3. Demographic reconstruction of North American and European *D. pulex* species.** *eSMC* and *MSMC2* were used to estimate historic effective population size (*N_e_*) output for each multi-locus genotype sample. We ran *eSMC* with a variable number of parthenogenic generations (G) per year from 5 to 10. Each multi-locus genotype sample was run independently. The shaded ribbon shows the upper 95% and lower 5% quantiles from the estimates. The bold line is the mean estimate.

**Supplemental Figure 4.** **Blue wavelength opsin gene and orthologous proteins in Crustacea and within-species heterozygosity. A)** This neighbor-joining protein tree was generated using *Blast’s* tree widget. The query sequence is highlighted in yellow and has a blue tip symbol. The green tip symbols are related Crustacean protein sequences with the species name in brackets. **B)** We subsampled two representative individuals within the European (EU) and North American (NA) *D. pulex* species and are showing the coverage for each individual set to [0-50]. The vertical-colored bars are heterozygous regions (i.e., split-colored bars) and homozygous alternative alleles (i.e., whole-colored bars), gray base pairs are the reference allele.

**Supplemental Figure 5.** **Segregation patterns and *F_IS_* of polymorphisms across lab and wild crossed *Daphnia* clones. A-C)** Average segregation frequency of F1 genotypes expected based on a double heterozygous cross (i.e., AB x AB) using empirical read depth at each SNP. We produced crosses of AxC in the lab shown in panel A and CxC shown in panel B. Panel C shows the F1 genotypes subsampled based on their status belonging to superclones identified in Barnard-Kubow et al. 2022, reflecting a conservative sampling approach. “Genome-wide” is the segregation for SNPs based on the read depth. “HWE” is the segregation pattern expected for Hardy Weinberg equilibrium. “Sim. BLOP” is the segregation pattern expected for the SNPs within the blue opsin gene based on empirical read depth. “BLOP” is the empirical segregation of trans-specific polymorphisms within the blue wavelength opsin gene among F1 genotypes. **D-F)** Distribution of average gene *F_IS_*. “HWE Simulation w/RD” is the expected *F_IS_* for each gene based on the empirical read depth for each SNP within every gene and “Empirical” is the average of the *F_IS_* across genes. The small arrow denotes where the gene average for the blue wavelength opsin falls along the empirical distribution.
